# Biomass smoke COPD has less tomographic abnormalities but worse hypoxemia compared with tobacco COPD

**DOI:** 10.1590/1414-431X20198233

**Published:** 2019-04-25

**Authors:** A.C. Meneghini, M. Koenigkam-Santos, M.C. Pereira, P.R. Tonidandel, J. Terra-Filho, F.Q. Cunha, M.B. de Menezes, E.O. Vianna

**Affiliations:** 1 Departamento de Medicina Social, Faculdade de Medicina de Ribeirão Preto, Universidade de São Paulo, Ribeirão Preto, SP, Brasil; 2 Departamento de Clínica Médica, Faculdade de Medicina de Ribeirão Preto, Universidade de São Paulo, Ribeirão Preto, SP, Brasil; 3 Departamento de Clínica Médica, Faculdade de Ciências Médicas, Universidade Estadual de Campinas, Campinas, SP, Brasil; 4 Departamento de Farmacologia, Faculdade de Medicina de Ribeirão Preto, Universidade de São Paulo, Ribeirão Preto, SP, Brasil

**Keywords:** Chronic obstructive pulmonary disease, Biomass, Tobacco

## Abstract

Special attention has emerged towards biomass smoke-induced chronic obstructive pulmonary disease (COPD), providing new knowledge for prevention and therapeutic approach of non-smoker COPD patients. However, the understanding of biomass smoke COPD is still limited and somewhat controversial. The aim of the present study was to compare COPD exclusively caused by tobacco smoking with COPD exclusively caused by environmental or occupational exposures. For this cross-sectional study, COPD patients were recruited from outpatient clinics and formed two groups: non-smoker COPD group (n=16) with exposure to biomass smoke who did not smoke cigarette and tobacco smoker COPD group (n=15) with people who did not report biomass smoke exposure. Subjects underwent pulmonary function tests, thoracic high-resolution computed tomography, 6-min walk test, and sputum induction. The non-smoker COPD group had biomass smoke exposure of 133.3±86 hour-years. The tobacco COPD group smoked 48.5±27.4 pack-years. Women were 62.5 and 66.7%, respectively, of non-smokers and smokers. The non-smoker COPD group showed higher prevalence of dyspnea, lower arterial oxygen tension (PaO_2_), and lower arterial oxygen saturation (SaO_2_%) with similar spirometry results, lung volumes, and diffusion capacity. Regarding inflammatory biomarkers, differences were detected in sputum number of lymphomononuclear cells and in sputum concentrations of interleukin (IL)-6 and IL-8 with higher values in the smoker group. Emphysema was more prevalent in the tobacco smoker group, which also showed higher relative bronchial wall thickness and lower lung density by quantitative analysis. Biomass smoke induced more hypoxemia compared to tobacco in COPD patients with similar severity.

## Introduction

Chronic obstructive pulmonary disease (COPD) is a public health issue for lower income countries as well as for the affluent world due to its increasing prevalence and mortality (1). Women are a particular concern of public authorities dealing with COPD epidemiological trends. Tobacco smoking is the leading cause of COPD. Biomass smoke exposure encompasses various biofuels used to heat or cook, such as wood, charcoal, grass, crop residues, animal dung, and dry leaves and twigs (2). Recently, special attention to causes of COPD other than tobacco smoking has emerged. This effort has provided knowledge to prevent COPD and to approach patients with COPD of different etiologies. For example, the role of chimneys on disease prevention and women susceptibility for non-tobacco COPD have been brought up for discussion in the last decades (3).

An increasing body of evidence has linked biomass smoke COPD to the predominance of the bronchial phenotype instead of the emphysema phenotype, when compared to tobacco-induced COPD (4–12). However, in a recent publication, Zhao et al. proposed a different phenotype by highlighting features of small airway disease (13). The authors found that biomass smoke COPD and tobacco COPD were associated with similar reductions in post-bronchodilator airflow obstruction, but biomass smoke COPD subjects had greater reductions in mid-expiratory flow, less pronounced markers of emphysema and, in endobronchial biopsies, greater intimal proliferation and basement membrane thickening (13).

This debate raised the possibility of a disease that is not COPD, or that is a “variant” of COPD (14). Regardless of the terminology, Han has stressed the need for considering ethnic differences, female susceptibility, and type and degree of exposure. The need for more studies was also emphasized: “While data regarding biomass smoke COPD is accumulating, how the phenotype of biomass smoke-related COPD compares to tobacco smoke-related COPD is not completely understood” (15).

We hypothesized that COPD associated with biomass burning smoke has different mechanisms and clinical and imaging profiles compared with tobacco-induced COPD. The aim of the present study was to compare COPD exclusively caused by tobacco smoking with COPD exclusively caused by environmental or occupational exposures.

## Material and Methods

### Subjects and protocol

This was a cross-sectional study. Patients were recruited from outpatient clinics in a University Hospital of Ribeirão Preto, São Paulo, Brazil. The city of Ribeirão Preto is a regional center in the Northeastern region of the São Paulo state, in Southeastern Brazil, with strong rural activity. The main economic activities are the sugar cane industry, trading, services, and financing. Data collection was done between 2015 and 2018.

Spirometric evaluation was performed for patient screening. COPD was defined according to GOLD criteria, i.e., post-bronchodilator VEF1/CVF <0.7 and history of exposure to etiologic factors (16). Most patients were aware of COPD diagnosis and treated accordingly. Following COPD diagnosis, patients were interviewed and a standardized questionnaire was applied to identify COPD risk factors.

### Groups

Patients were enrolled into two groups: non-smoker group or tobacco smoker group. Subjects reporting both tobacco smoking and significant environmental exposures were excluded from this study. Therefore, only COPD patients who reported a specific exposure, tobacco or environmental, were included in this study protocol.

Smoker COPD patients with exposure to other etiologic factors were excluded. Other exclusion criteria were pulmonary diseases other than COPD; any evidence of asthma or asthma symptoms in childhood or adolescence; respiratory symptoms that preceded exposure; and bronchiectasis, tuberculosis, fungal lung disease, and cystic fibrosis. In order to rule out these diseases, patients were evaluated by measuring immunoglobulin G (IgG), alpha-1 antitrypsin, IgA, and sweat chloride. Patients reporting use of systemic corticosteroids or respiratory infections in the previous 12 weeks were not included.

### Ethics procedures

Information regarding the study was offered and a consent form was signed by every subject before any study procedure. Both the project and the consent form were previously approved by the Institutional Review Board.

### Pulmonary function tests

Spirometry and diffusion lung capacity measurements were performed by using a GS Plus (Collins, USA). Maneuvers and efforts followed the American Thoracic Society recommendations. Subjects performed the test in a seated position, wearing nose clips, and using disposable mouthpieces. The predictive values were based on international reference equations (17–19). For the bronchodilator test, patients inhaled 400 mcg of albuterol from a metered dose inhaler. Arterial blood was collected preferably from the radial artery. The collected sample was analyzed immediately. Reference values followed ATS standardization (20).

### High-resolution chest computed tomography

All patients were examined within a multidetector computed tomography scanner and images were acquired using a high-resolution (HRCT) protocol: volumetric acquisitions without intravenous contrast media, craniocaudal direction, supine position, at full inspiration, and relaxed exhalation after coaching. Typical parameters were: 1 mm slice thickness, 150–200 mAs, 120 kVp tube current, 0.5 second rotation time, and radiation exposure lower than 5 mSv. Images were reconstructed with 1 mm intervals using soft (standard) and hard reconstruction kernels, and could be displayed in lung or mediastinal windows.

Subjective imaging analysis was done by one experienced thoracic radiologist, blinded to the patients’ clinical data, who identified the following characteristics on HRCT: presence or absence and degree (mild, moderate, severe) of emphysema, types of emphysema (centrilobular, panlobular, paraseptal), presence or absence of airway disease (wall thickening, irregularity, airway opacities), presence or absence of air trapping on expiratory images, and presence or absence of bronchiectasis.

Quantitative analysis of HRCT scans was done with Yacta academic software version 2.7 (21). Yacta is a fully automated computer program that segments (anatomically separates) the airways, blood vessels, right and left lungs, and pulmonary lobes, using attenuation thresholds and anatomical recognition algorithms. It is able to provide lung mean volumes and densities, histograms, and relative values of lung parenchyma density, including percentiles. The program detects the areas of emphysema (low attenuation areas with a threshold of –950 HU) and allows the evaluation of the type and distribution of emphysema. Yacta also performs the airways analysis, with the three-dimensional evaluation of the whole tracheobronchial tree, obtaining measures of caliber, wall thickness, and derived parameters of the bronchi, using different algorithms.

In this study, we analyzed the lung volume (cm^3^), emphysema volume (cm^3^) and index (emphysema/lung volumes, in %), lung mean density (HU), relative bronchial wall thickness (to bronchus diameter, in %) for 3rd to 8th bronchi generation (excluding trachea and main bronchi), Pi10 (normalized measurement of wall thickness for a standardized 10 mm diameter bronchus), and the number of measured bronchi (which correlates with bronchodilation and presence of bronchiectasis) (21).

### Six-min walk test

The six-min walk test (6MWT) is a submaximal exercise test that allows evaluation of several lung diseases (22). The protocol was performed in a walking course with 30 m of length. At every min, we performed assessment of dyspnea by the Borg index, SpO2, and heart rate for 6 min (23).

### Sputum induction

The guidelines of the Task Force on Induced Sputum of the European Respiratory Society were applied (24 [Bibr B25]). Sputum induction was performed by inhalation of a hypertonic saline (4.5% NaCl) aerosol delivered by an ultrasonic nebulizer (Ultra-Neb 2000; DeVilbiss, USA). The inhalation duration was 20 min, divided into four 5-min periods. For safety reasons, a peak expiratory flow (PEF) measurement was performed every 5 min and the procedure was stopped if the PEF fell to the critical value (a 10% fall from the basal value) or if there were bothersome symptoms. During the induction, the subjects were encouraged to spit saliva into a plastic container and sputum into another pre-weighed sterile container anytime they wished. Saliva was discarded, the sputum was weighed and 1 mL of 0.1% dithiothreitol (DTT; Gibco BRL, USA) per gram of sputum was added. The suspension was shaken in a vortex mixer for a few seconds and incubated in a shaking water bath (Dubnoff TE-053; TECNAL, Brazil) at 37°C (150 cycles/min) for 15 min, with periodic brief aspirations. The sample was centrifuged at 750 *g* for 10 min at 25°C (Allegra 21R centrifuge; Beckman, USA) and the supernatant was aspirated and stored at –85°C for measurement of cytokines by ELISA test.

### Questionnaires

Questionnaires were employed for dyspnea evaluation, socioeconomic status characterization, COPD severity assessment, other symptoms, and exposure evaluation (22–26).

### Statistical analysis

The between-group comparison of frequency of outcomes was performed by Fisher's exact test. The non-parametric Wilcoxon test was used to compare continuous variables between groups. Multiple linear regression analysis was performed to test the effect of group and age on arterial oxygen tension (PaO_2_). Correlations between variables were tested by calculating Pearson's correlation coefficient. Tests were performed using the computer program R Core Team (2016). R is a language and environment for statistical computing (R Foundation for Statistical Computing, Vienna, Austria. <https://www.R-project.org>).

## Results

Thirty-one subjects completed the protocol, 16 in the non-smoker COPD group and 15 in the smoker COPD group. The non-smoker COPD group had biomass smoke exposure of 133.3±86 hour-years and 93.7% lived in rural locations in the past. All subjects in this group had domestic biomass smoke exposure. For the smoker COPD group, 46.7% had lived in rural locations in the past. Both urban and rural locations were in the same region of Brazil, with similar climate and altitude. The smoker COPD group had tobacco smoking history of 48.5±27.4 pack-years. Second-hand tobacco smoke exposure was reported by 81.2 and 53.3%, respectively, in the non-smoker and smoker patients.

The biomass smoke group was significantly older (P<0.01), surpassing 10 years. Women were the majority among non-smokers and smokers, 62.5 and 66.7%, respectively. COPD severity was not different between groups and there was no difference in the walked distance during the 6MWT (Table 1).

**Table 1.** Patient characteristics according to chronic obstructive pulmonary disease (COPD) groups.

Variable	Non-smoker group (n=16)	Tobacco smoker group (n=15)	P value
Age (years)	76.1±7.1	63.9±6.6	<0.01
Female (%)	10 (62.5%)	10 (66.7%)	>0.50
BMI (kg/m^2^)	26.5±5.6	28.5±6.2	0.30
CAT score			
Mild	4 (25.0%)	4 (26.7%)	>0.50
Moderate	6 (37.5%)	6 (40.0%)	
Severe	4 (25.0%)	3 (20.0%)	
Very severe	2 (12.5%)	2 (13.3%)	
Dyspnea mMRC scale			
Grade 0	4 (25.0%)	4 (26.7%)	0.22
Grade 1	6 (37.5%)	6 (40.0%)	
Grade 2	4 (25.0%)	3 (20.0%)	
Grade 3	2 (12.5%)	2 (13.3%)	
COPD severity			
Stage I (mild)	5 (31.2%)	2 (13.3%)	0.25
Stage II (moderate)	7 (43.7%)	8 (53.3%)	
Stage III (severe)	2 (12.5%)	5 (33.3%)	
Stage IV (very severe)	2 (12.5%)	0	
COPD classification			
GOLD A	3 (18.7%)	3 (20.0%)	>0.50
GOLD B	6 (37.5%)	5 (33.3%)	
GOLD C	1 (6.2%)	1 (6.7%)	
GOLD D	6 (37.5%)	6 (40.0%)	
6MWT (m)	263±93	297±73	0.30

Data are reported as means±SD or number and percentage. Statistical analysis was done with Fisher's exact test or Wilcoxon test. BMI: body mass index; mMRC: Modified Medical Research Council (23); GOLD A: less symptoms and low risk; GOLD B: more symptoms and low risk; GOLD C: less symptoms and high risk; GOLD D: more symptoms and high risk (GOLD, 2011); 6MWT: 6-min walk test.

The non-smoker COPD group showed a higher prevalence of dyspnea, lower PaO_2_ and lower arterial oxygen saturation (SaO_2_%) with similar spirometry results, lung volumes, and diffusion capacity (Table 2).

**Table 2.** Respiratory symptoms and pulmonary function tests according to chronic obstructive pulmonary disease groups.

Variable	Non-smoker group	Tobacco smoker group	P value
Respiratory symptoms			
Cough	11 (69.0%)	8 (53.3%)	1.00
Sputum	6 (37.5%)	6 (40.0%)	1.00
Morning throat	7 (44.0%)	7 (46.7%)	1.00
Wheezing	6 (37.5%)	5 (33.3%)	1.00
Dyspnea	13 (81.2%)	5 (33.3%)	0.01
Post-bronchodilator spirometry			
FVC, %predicted	75.7±18.1	83.0±14.1	0.20
FEV_1,_ %predicted	59.8±22.4	59.5±16.7	0.90
FEV_1_/FVC	0.53±0.1	0.52±0.1	0.70
Lung volumes			
TLC, %predicted	107.3±22.7	112.6±14.0	0.50
RV, %predicted	156.9±45.5	170.7±38.1	0.40
DLCO			
DSB (mL/min/mmHg)	11.8±5.0	13.6±7.4	0.40
DSB, %predicted	62.6±25.5	63.3±27.9	0.90
Arterial blood gases			
PaO_2_ (mmHg)	66.1±8.5	74.8±7.9	0.01
Age-adjusted PaO_2_ (mmHg)	74.9±5.0	79.5±5.4	0.02
PaCO_2_ (mmHg)	42.6±7.7	40.7±5.1	0.40
pH	7.4	7.4	1.00
SaO_2_%	92.5±4.1	95.4±2.0	0.02

Data are reported as means±SD or number and percentage. Statistical analysis was done with Fisher's exact test or Wilcoxon test. FVC: forced vital capacity; FEV_1_: forced expiratory volume in one second; TLC: total lung capacity; RV: residual volume; DLCO: diffusing capacity of the lung for carbon monoxide; DSB: carbon monoxide diffusion capacity; PaO_2_: arterial oxygen tension; PaCO_2_: arterial carbon dioxide tension; SaO_2_%: arterial oxygen saturation.

Multiple linear regression analysis was employed to evaluate the effect of both age and group on PaO_2_. Therefore, PaO_2_ was the dependent variable and the independent variables were group and age. Results are shown in Table 3 and allowed us to notice that group (non-smoker group) had significant effect on PaO2 in both simple and adjusted regression models. Tobacco smoker group effect increased PaO_2_ with a coefficient of 9 and an intercept estimated coefficient of 64 and P=0.01. This effect was unrelated to age, and age had a non-significant effect on PaO_2_.

**Table 3.** Multiple linear regression analysis of the effect of group and age on arterial oxygen tension (PaO_2_).

Variable	Simple regression model				Multiple regression model			
	Estimated coefficient	Standard error	P value	R^2^	Estimated coefficient	Standard error	P value	R^2^
Tobacco smoker group	8.70	3.00	0.01	0.22	9.00	4.08	0.04	0.22
Age	–0.31	0.20	0.09	0.09	0.03	0.22	0.90	0.22

PaO_2_: arterial oxygen tension.

The Pearson correlation test was used to test the correlation between PaO_2_ and diffusion capacity (r=0.20; 95% confidence interval: −0.17; 0.53); between PaO_2_ and volume of emphysema, r=0.05 (−0.3; 0.4); between PaO_2_ and emphysema index, r=−0.13 (−0.5; 0.2); between lung mean density and age, r=0.30 (−0.06; 0.59); between emphysema index and PaO_2_, r=−0.13 (−0.47; 0.23); between diffusion capacity and age, r=−0.10 (−0.45; 0.27); between volume of emphysema and age, r=−0.34 (−0.62; 0.01); between emphysema index and age, r=−0.2 (−0.58; 0.08); between volume of emphysema and diffusion capacity, r=−0.31 (−0.6; 0.06); between emphysema index and diffusion capacity, r=−0.25 (−0.57; 0.13); and between lung mean density and diffusion capacity, r=−0.06 (−0.4; 0.3).

Data on sputum cytology, and sputum and blood cytokines are shown in Table 4. Differences were detected in sputum lymphomononuclear cells number and in sputum concentrations of interleukin (IL)-6 and IL-8 with increased values in the smoker group.

**Table 4.** Inflammatory biomarkers according to chronic obstructive pulmonary disease groups.

Variable	Non-smoker group	Tobacco smoker group	P value
Sputum cytology			
Weight (g)	3.5±2.0	3.2±3.1	0.80
Number of cells (x10^6^)	17.8±17.9	6.1±6.1	0.04
Viable cells (%)	83.1±13.6	79.6±13.6	0.50
Squamous cells (%)	16.3±24.6	11.6±13.8	0.50
Neutrophils (%)	79.6±14.0	69.3±15.0	0.07
Eosinophils (%)	4.2±4.4	4.8±7.3	0.80
Lymphomononuclear cells (%)	16.1±12.6	25.9±12.9	0.04
Sputum biomarkers			
TNF-α (pg/mL)	268.3±563.5	153.7±180.0	0.50
IL-6 (pg/mL)	174.1±229.1	1027.7±1282.6	0.03
IL-8 (pg/mL)	1445.1±1325.9	3055.9±1914.0	0.04
Blood biomarkers			
TNF-α (pg/mL)	21.3±47.5	46.0±8.0	0.30
IL-6 (pg/mL)	66.5±114.4	49.8±84.5	0.60
IL-8 (pg/mL)	163.6±470.2	334.0±789.8	0.50

Data are reported as means±SD or number and percentage. Statistical analysis was done with Fisher's exact test or Wilcoxon test. IL: interleukin; TNF: tumor necrosis factor.

Table 5 shows data on HRCT. Emphysema was more prevalent in the tobacco smoker group according to the qualitative analysis. This group had higher relative bronchial wall thickness from the 3rd to 8th bronchial division and lower lung density as assessed by quantitative analysis. Seven patients were above the cut-off value of 6% for emphysema index in the smoker group and 2 patients were above it in the non-smoker group.

**Table 5.** High resolution computed tomography findings according to chronic obstructive pulmonary disease groups.

Variable	Non-smoker group	Tobacco smoker group	P value
Descriptive evaluation (thoracic radiologist)			
Emphysema	4 (25%)	12 (80.0%)	<0.02
Centrilobular emphysema	4 (25%)	12 (80.0%)	<0.01
Paraseptal emphysema	2 (12.5%)	7 (46.7%)	>0.05
Panlobular emphysema		2 (13.3%)	
Air trapping	4 (25.0%)	3 (20.0%)	>0.50
Bronchiectasis	7 (43.7%)	2 (13.3%)	>0.05
Quantitative evaluation (Yacta software)			
Lung volume (cm^3^)	4368.2±1358.2	5152.8±1124.8	0.09
Volume of emphysema (cm^3^)	173.3±814.6	577.1±641.6	0.09
Emphysema index (%)	2.7±4.4	17.5±28.7	0.07
Lung mean density (HU)	-787.3±4.4	-821.3±32.8	0.02
Relative bronchial wall thickness 3rd-8th generation (%)	31.6±26.9	52.7±15.8	0.01
Pi10	0.34±0.1	0.44±0.25	0.18
Measured bronchi (n)	25.7±14.1	24.8±24.8	0.90

Data are reported as means±SD or number and percentage. Statistical analysis was done with Fisher's exact test or Wilcoxon test. HU: Hounsfield units; Pi10: normalized thickness of the bronchial wall.

## Discussion

In our study, the non-smoker COPD group had a higher prevalence of dyspnea, lower PaO_2_ and SaO_2_, and lower concentrations of IL-6, IL-8, and mononuclear cells in sputum. HRCT of these patients showed a predominant airway disease phenotype of COPD, with less emphysema (qualitatively and quantitatively evaluated), higher mean lung density, and less severe bronchial wall thickening compared with smoker COPD. Lung function results, including diffusion capacity measurements, were not different between groups.

We showed that hypoxemia was associated with biomass smoke-induced COPD. Although emphysema was more prevalent in the smoker group, lung diffusion capacity was similarly reduced in both groups, mean of 63% of predicted value, which at least partially rules out emphysema as a putative explanation for differences in PaO_2_ and SaO_2_% between etiologies. Camp et al. (11) described low O_2_ saturation that was not confirmed by reduced PaO_2_ in biomass smoke COPD compared with smoker COPD. Moreover, in that study, PaCO_2_ was reduced in the smoker group and this hyperventilation could have increased O_2_ saturation. Their subjects were not as severe as those included in the current study, and they hypothesized that low O_2_ saturation could be a consequence of high altitudes in Mexico City, where their study was done. Therefore, our study offers a new step forward to confirm hypoxemia and to associate it with dyspnea, which was higher in the non-smoker subjects (11).

Age can influence PaO_2_ and this could be a confounder in our study, since non-smoker group subjects were older with lower PaO_2_. In order to evaluate the effect of age, two approaches were employed. First, we calculated real values of PaO_2_ according to age in every subject by the ATS formula (20). Second, we controlled PaO_2_ for age by multiple linear regression analysis. In any case, PaO2 was significantly lower in the biomass smoke COPD group.

IL-6 and IL-8 are associated with tobacco-induced COPD. These biomarkers have been screened as potential diagnostic tools (27). The concentrations of these cytokines were higher in sputum, but not in blood, in the smoker group compared to the biomass smoke group. Meanwhile, there was an increased number of sputum mononuclear cells (lymphocytes and macrophages) in the smoker group. Similar to the increase of IL-6 and IL-8 associated to cigarette smoking, this may be interpreted as increased inflammation. Zhao et al. (13) compared early cases of biomass smoke COPD with cigarette smoke COPD. Patients underwent bronchoalveolar lavage that revealed increased numbers of macrophages and lymphocytes in biomass smoke COPD. The severity of their patients or the lung compartment sampled by bronchoalveolar lavage versus sputum may be the explanation for these contradictory results.

A strength of our selection criteria is the lack of group overlap, which allowed us to detect small differences between groups. Conversely, this characteristic was also a limitation, since some studies suggest that biomass smoke may interact with cigarette smoking in the pathogenesis of COPD (2,28) and we cannot make any assumption regarding interaction of these two risk factors. The similar proportion of women in both groups is another advantage of this sample. Another study limitation is the sample size that is also related to the difficulty of recruiting only smokers or only non-smokers.

The asthma-COPD overlap syndrome has been neglected in some studies on biomass smoke COPD. Since asthma is a phenotype with no emphysema, with predominance of bronchial symptoms, and bronchial thickening in radiology, it is necessary to assess and exclude asthmatic patients from the sample of non-smoker COPD to describe this disease correctly. We made different attempts to exclude asthmatic patients, even in the case of overlap syndrome. Thus, we excluded subjects with a history of asthma or clinical features resembling asthma (like symptoms that preceded longtime exposure). As evidence of successfully detecting and excluding asthma patients, data showed no difference in the number of eosinophils in blood or sputum and no difference in bronchodilator response between groups.

This study design was not conceived to compare severity or time-course between COPD etiologies. We have included hospital-defined COPD cases confirmed by spirometry. By selecting cases from the public health system, patients were not representative of the general population; more severe cases might predominate herein. In addition, either risk factor may induce COPD earlier than the other one.

Most phenotype discussions in previous articles on COPD associated with biomass smoke have been based on the dichotomous COPD view focused on emphysema and chronic bronchitis. Interestingly, data provided by this study with CT scans, pulmonary function, and inflammatory biomarkers revealed that tobacco induced-COPD was more severe in emphysema and bronchitis. On the other hand, biomass smoke induced more hypoxemia and dyspnea compared to tobacco in COPD patients with similar spirometry severity. This phenotype may be associated with ventilation-perfusion mismatch leading to hypoxemia with less visible damage to lung parenchyma and to the bronchial compartment assessed by CT scans. We certainly need to further study this phenotype to better understand mechanisms of hypoxemia and consequences in terms of prognosis and therapy requirements.
